# Double Trouble: Clinical and Psychological Characteristics in SCAD With and Without Fibromuscular Dysplasia

**DOI:** 10.1016/j.jacadv.2026.102814

**Published:** 2026-06-17

**Authors:** Lisa-Marie Maukel, Laurie-Anne Boivin-Proulx, Jacqueline Saw, Mina Madan, Shuangbo Liu, Thais Coutinho, Sharon Mulvagh, Christine Pacheco, Karen Bouchard, Jennifer Reed, Louise Sun, Amélie Paquin, Nadia Lappa, Derek So, Heather Tulloch

**Affiliations:** a University of Ottawa Heart Institute, Ottawa, Ontario, Canada; b University of British Columbia, Vancouver, British Columbia, Canada; c University of Toronto, Toronto, Ontario, Canada; d University of Manitoba, Winnipeg, Manitoba, Canada; e Mayo Clinic, Rochester, Minnesota, USA; f Dalhousie University, Halifax, Nova Scotia, Canada; g Université de Montréal, Montréal, Quebec, Canada; h Hôpital Pierre-Boucher, Centre de santé et de Services Sociaux de la Montérégie-Est, Montreal, Quebec, Canada; i University of Ottawa, Ottawa, Ontario, Canada; j Stanford University School of Medicine, Stanford, California, USA; k Laval University, Quebec City, Quebec, Canada; l Patient Partner of the University of Ottawa Heart Institute, Ottawa, Ontario, Canada

**Keywords:** fibromuscular dysplasia, FMD, mental health, psychological distress, SCAD, spontaneous coronary artery dissection

## Abstract

**Background:**

Spontaneous coronary artery dissection (SCAD), a cause of acute coronary syndrome predominantly affecting women, is associated with substantial psychological distress. Fibromuscular dysplasia (FMD), a nonatherosclerotic vascular disorder frequently associated with SCAD, adds clinical complexity and may exacerbate distress. However, differences in patient characteristics between SCAD patients with and without FMD remain poorly studied.

**Objectives:**

The objective of the study was to compare demographic, clinical, and psychological characteristics of SCAD patients with and without FMD.

**Methods:**

In this multicenter study, SCAD patients diagnosed <3 years prior underwent chart review for medical data, including FMD screening. Participants completed sociodemographic questionnaires and validated psychological measures: Patient Health Questionnaire-9 (depressive symptoms), Generalized Anxiety Disorder-7 (generalized anxiety), Cardiac Anxiety Questionnaire (cardiac anxiety), and Post-traumatic Stress Disorder Checklist-5 (post-traumatic stress). Between-group differences were analyzed using t-tests and chi-square tests.

**Results:**

Of 326 participants, 296 (90.8%) completed FMD screening, with 128 (43.2%) meeting the diagnostic criteria. Compared to those without FMD, patients with FMD were older, more often females, and more likely to have a history of migraines. Among 67 patients aware of their FMD diagnosis, psychological distress was higher than in those without known FMD: depression 32.8% vs 20.8%, anxiety 31.3% vs 17.4%, cardiac anxiety 64.2% vs 49.2%, and post-traumatic stress 25.4% vs 11.6% (all *P* < 0.05).

**Conclusions:**

SCAD patients who receive a concomitant FMD diagnosis exhibit distinct characteristics and substantially higher psychological distress, highlighting the need for early, comprehensive screening for both FMD, and psychological distress. Given that FMD diagnosis itself may increase psychological burden, anticipatory psychological support and tailored interventions are warranted for this high-risk subgroup.

Spontaneous coronary artery dissection (SCAD) is an increasingly recognized cause of acute coronary syndrome (ACS), particularly among younger and middle-aged women.^1^ It results from a separation of the layers of the coronary artery wall and the formation of an intramural hematoma.^2^ Typically, SCAD occurs in the absence of atherosclerosis, trauma, or iatrogenic causes.^1^ Unlike other causes of ACS, traditional cardiovascular risk factors are less prevalent in individuals with SCAD.^3^ The unpredictable nature of the condition, the frequent persistence of anginal symptoms even after vessel healing, and the potential for recurrence^4^ all contribute to the substantial psychological distress experienced by patients with SCAD.^5^, ^6^, ^7^, ^8^

Fibromuscular dysplasia (FMD) is a nonatherosclerotic, noninflammatory arteriopathy affecting medium-sized arteries, most commonly the carotid, coronary, and renal arteries, leading to stenosis, aneurysm formation, or dissection.^9^ FMD is commonly observed in patients with SCAD, although routine screening early after SCAD is unstandardized and center-dependent.^1^ Reported prevalence varies widely (17% to 86%),^10^^,^^11^ with studies using rigorous comprehensive screening, such as catheter-based angiography of the bilateral iliofemoral and renal arteries, often reporting >70%.^10^^,^^12^ The coexistence of SCAD and FMD suggests a potential shared vascular pathology, highlighting the need to better understand how this comorbidity influences clinical presentation and outcomes.

Few studies^12^, ^13^, ^14^ have examined demographic, clinical, and psychological differences between SCAD patients with and without FMD. In the Vancouver SCAD Registry (n = 346; 250 with FMD), patients with extra coronary FMD were older and more likely to have a history of myocardial infarction, stroke, or transient ischemic attack.^12^ Similarly, in the French DISCO Registry of patients with SCAD (n = 340, 153 with FMD), those with concurrent FMD were older, more likely to have hypertension, and more often postmenopausal.^14^ In a small study of 51 women with SCAD, those with FMD were older, more frequently presented with type 2 SCAD, and were more likely to have tortuous coronary arteries and additional vascular abnormalities.^13^ Obstetric and gynecological characteristics in association with comorbid FMD status remain largely unexplored and very little is known about mental health among patients with SCAD and FMD. To our knowledge, only one small study (n = 10) examining mental health symptoms among these groups exists. Higher rates of depression or anxiety were observed among individuals with both SCAD and FMD,^13^ suggesting that having 2 serious vascular diagnoses may be associated with increased psychological distress. Taken together, there is a paucity of data regarding clinical and psychological correlates among patients with concomitant SCAD and FMD.

The multisite Canadian MINDSET study was designed to characterize psychological distress in patients with SCAD. Although all participants were aware of their confirmed SCAD diagnosis, not all had been screened and/or informed of a concomitant FMD diagnosis. This setting allowed us to examine whether awareness of an additional vascular condition is associated with increased psychological distress. The present analysis aimed to: 1) determine the prevalence of patients with SCAD and FMD by angiography; 2) compare clinical characteristics and management of SCAD patients with and without a concomitant diagnosis of FMD; and 3) examine differences in psychological distress between patients with SCAD who are aware or not aware of a concomitant FMD diagnosis.

## Methods

### Study design and population

Eligible participants were adults (age ≥18 years) with a confirmed SCAD diagnosis by angiography within the past 3 years, recruited from 6 Canadian hospitals between April 2022 and January 2025. Recruitment was conducted by phone or in person at the bedside, in cardiology clinics, including interventional cardiology follow-ups, Women’s Heart Clinic appointments, and through cardiac rehabilitation programs. Participants had the option to complete questionnaires on paper, either on-site or at home, or electronically through the secure REDCap platform.^15^

This study received ethical approval from all participating sites (Ottawa Health Science Network Research Ethics Board; Protocol ID: 20210797-01H). Informed consent was obtained from all participants included in the study.

### Measurements

#### Demographics and medical information

Participants completed a questionnaire collecting demographic information (eg, age, sex, and ethnicity) and self-reported medical history, including pre-SCAD mental health professional contacts, pre-SCAD mental health diagnoses, and potential precipitating factors for SCAD. All other clinical data, including obstetric and gynecological history (eg, preeclampsia, gestational hypertension, premature birth, stillbirth/fetal demise, abnormal uterine bleeding, and menopausal hormonal therapy), were documented by the treating physician and subsequently abstracted via standardized chart review by research coordinators at each participating site under the supervision of the site principal investigator.

#### Ascertainment of FMD diagnosis (angiography and self-report)

FMD screening was not a mandatory component of this SCAD study and was performed at the discretion of treating physicians. However, all participating sites aim to systematically screen patients with SCAD for FMD. A diagnosis of FMD was made if peripheral diagnostic angiography (at time of cardiac catheterization), computed tomography (CT) or, magnetic resonance (MR) angiography in any of 3 vascular territories (cerebrovascular, renal, and iliac arteries) demonstrated the presence of multifocal lesions with the characteristic “string-of-beads” appearance, as documented in the medical record by the treating physician. Research coordinators at each participating site abstracted this data from medical chart at study completion. These verified FMD statuses were used for all analyses of clinical and procedural data.

Some patients did not have FMD screening completed or the screening results were not available at the time of psychological questionnaire completion. To ensure that the psychological distress comparison reflected awareness of an FMD diagnosis, we used self-reported FMD status at the time of questionnaire completion; participants who were aware of their diagnosis (screened and informed) were compared to those who were not aware (either no FMD or not yet screened/informed).

#### Psychological distress measures

##### Depressive symptoms

Depressive symptoms were assessed using the Patient Health Questionnaire-9.^16^^,^^17^ Participants rated their symptoms over the past 2 weeks using 9 items on a 4-point Likert scale (0 = not at all; 3 = nearly every day), yielding total scores from 0 to 27. A cutoff score ≥10 reflects clinically significant depression.^16^ The tool demonstrates strong psychometric properties, with good internal consistency (Cronbach’s α = 0.89) and established validity.^16^

##### Anxiety symptoms

Anxiety symptoms were measured using the Generalized Anxiety Disorder-7 (GAD-7) scale.^18^ Participants rated their symptoms over the past 2 weeks using 7 items on a 4-point Likert scale (0 = not at all, 3 = nearly every day), yielding total scores from 0 to 21. Scores ≥10 indicate clinically significant anxiety.^18^ In cardiac patients, the GAD-7 demonstrated strong reliability (α = 0.89; composite reliability = 0.90) and validity across procedural, criterion, construct, and factorial measures.^19^

##### Cardiac anxiety

Heart-focused anxiety was assessed using the Cardiac Anxiety Questionnaire (CAQ), an 18-item scale evaluating 3 subdomains: fear of cardiac-related sensations, heart-focused attention, and avoidance of activities eliciting cardiac symptoms.^20^ Subscale and total scores were computed, with higher scores indicating greater cardiac anxiety.^20^ Based on the median of this sample, scores of 26 or higher were considered clinically significant.^21^ The CAQ showed good internal consistency and construct validity.^20^^,^^22^

##### Post-traumatic stress symptoms

Post-traumatic stress symptoms were assessed with the Post-traumatic Stress Disorder (PTSD) Checklist-5 (PCL-5),^23^^,^^24^ a 20-item self-report questionnaire aligned with The Diagnostic and Statistical Manual of Mental Disorders-5 PTSD criteria. Participants rated their symptoms over the past month in relation to their cardiac event using a 5-point Likert scale (0 = not at all; 4 = extremely), with total scores ranging from 0 to 80. Scores ≥31 indicate clinically significant PTSD symptoms and probable diagnosis.^24^ The PCL-5 demonstrates strong reliability and validity in assessing PTSD symptoms across diverse populations.^23^^,^^24^

### Statistical analysis

Data analyses were conducted using IBM SPSS (version 29). Missing data were minimal (0% to 3% per variable) and met the criteria for missing completely at random (Little’s Missing Completely at Random test: chi-square = 2,916.2; *P* = 0.658). Missing values in psychological questionnaire data were addressed via semiparametric multiple imputation^25^ (m = 5), using demographic (age, sex, ethnicity, and education) and clinical variables (time since diagnosis, pre-SCAD mental health professional contact, and pre-SCAD mental health diagnosis) as predictors. Clinical data were not imputed.

Descriptive statistics for demographic, clinical, and psychological variables were computed, with continuous variables reported as mean ± SD and categorical variables as frequencies and percentages. Psychological outcomes were analyzed both as continuous total scores and as binary variables indicating clinically significant distress based on established cutoffs. Group differences were analyzed using independent samples t-tests for continuous variables and chi-square tests for categorical variables.

Sensitivity analyses were performed in patients with angiographically confirmed FMD at study completion (N = 128), comparing those who were aware of their diagnosis with those who were unaware.

To preserve statistical power, multivariable linear regression analyses were conducted in the full sample (N = 326), examining the association between FMD awareness and mean scores of Patient Health Questionnaire-9, GAD-7, CAQ, and PCL-5, adjusting for sex, age, and pre-SCAD mental health diagnosis (yes vs no). Unstandardized regression coefficients (*B*) with 95% CIs, *t*-values, and model fit indices (*R*^*2*^, adjusted *R*^*2*^) were reported. Effect sizes were interpreted as small (*R*^*2*^ = 0.02), medium (*R*^*2*^ = 0.13), and large (*R*^*2*^ = 0.26).^26^ A 2-tailed *P* value <0.05 was considered statistically significant.

## Results

### Prevalence of FMD in the SCAD study cohort

A total of 326 patients with SCAD were included in the study (92.9% female, 84.4% White), with a mean age of 53.3 ± 11.1 years and an average of 15.9 ± 12.5 months since their SCAD (Table 1). FMD screening results were not available for 30 patients (9.2%) at the time of study closure: 26 (8.0%) had not yet undergone screening and 4 (1.2%) were awaiting results. Of the remaining 296 patients (90.8%) who received FMD screening by study completion, 128 (43.2%) met the diagnostic criteria for FMD.

**Table 1:** Demographic and Clinical Characteristics in SCAD With vs Without FMD

	Overall (N = 296)	FMD (n = 128)	No-FMD (n = 168)	*P* Value
Age, mean (SD)	53.1 (11.2)	54.9 (9.9)	51.7 (12.0)	0.015
Time since diagnosis in months, mean (SD)	15.7 (12.5)	17.3 (13.2)	14.4 (11.8)	0.044
Female, n (%)	276 (93.2)	126 (98.4)	150 (89.3)	0.002
Smoking, n (%)	19 (6.4)	9 (7.0)	10 (6.0)	0.718
Dyslipidemia, n (%)	99 (33.4)	44 (34.4)	55 (32.7)	0.767
Hypertension, n (%)	83 (28.0)	37 (28.9)	46 (27.4)	0.772
Diabetes type II, n (%)	17 (5.7)	6 (4.7)	11 (6.5)	0.496
Coronary artery disease, n (%)	76 (25.7)	24 (18.8)	52 (31.0)	0.017
Peripheral artery disease, n (%)	1 (0.3)	1 (0.8)	0 (0.0)	0.432
Previous stroke, n (%)	6 (2.0)	3 (2.3)	3 (1.8)	1.000
Aortic aneurysm, n (%)	4 (1.4)	2 (1.6)	2 (1.2)	1.000
Previous aortic dissection, n (%)	1 (0.3)	0 (0.0)	1 (0.6)	1.000
Cerebral aneurysm, n (%)	2 (0.7)	2 (1.6)	0 (0.0)	0.186
Migraines, n (%)	47 (15.9)	27 (21.1)	20 (11.9)	0.032
Mental health condition pre-SCAD	84 (28.6)	43 (33.9)	41 (24.6)	0.080
Predisposing conditions, n (%)				
Pregnancy^a^	1 (0.4)	0 (0.0)	1 (0.7)	1.000
Postpartum^a^	14 (5.4)	5 (4.1)	9 (6.4)	0.412
Multiparity (≥4 births)^a^	16 (5.8)	7 (5.6)	9 (6.0)	0.875
Other connective tissue disorders	6 (2.0)	1 (0.8)	5 (3.0)	0.240
Systematic inflammatory disorders (SLE, RA, and PsA)	9 (3.0)	6 (4.7)	3 (1.8)	0.182
Vasospastic angina	11 (3.7)	4 (3.1)	7 (4.2)	0.762
Precipitating factors, n (%)				
Intense physical activity	85 (28.7)	41 (32.0)	44 (26.2)	0.271
Intense emotional stress	153 (51.7)	71 (55.5)	82 (48.8)	0.256
Labor/delivery^a^	7 (2.5)	1 (0.8)	6 (4.0)	0.130
Other	49 (16.6)	19 (14.8)	30 (17.9)	0.490
Clinical presentation, n (%)				0.918
STEMI	87 (30.3)	40 (32.3)	47 (28.8)	
NSTEMI	188 (65.5)	79 (63.7)	109 (66.9)	
Ventricular arrhythmia	3 (1.0)	1 (0.8)	2 (1.2)	
Other	9 (3.1)	4 (3.2)	5 (3.1)	
Paraclinical characteristics				
Left ventricular ejection fraction, mean (SD)	55.3 (8.8)	54.8 (9.1)	55.6 (8.5)	0.490
Diastolic blood pressure, mean (SD)	73.0 (11.3)	73.4 (10.9)	72.6 (11.6)	0.606
Systolic blood pressure, mean (SD)	119.7 (20.6)	120.1 (19.7)	119.7 (21.3)	0.885
Heart rate, mean (SD)	68.6 (12.5)	68.2 (12.7)	68.9 (12.4)	0.643
Procedural characteristics				
Type II SCAD classification, n (%)	144 (48.6)	66 (51.6)	78 (46.4)	0.381
Culprit artery, n (%)				0.370
LAD or branch	122 (41.5)	50 (39.4)	72 (43.1)	
Circumflex or branch	60 (20.4)	25 (19.7)	35 (21.0)	
>1	31 (10.5)	18 (14.2)	13 (7.8)	
Other	81 (27.6)	34 (26.8)	47 (28.1)	
Intravascular imaging, n (%)				
IVUS	8 (2.7)	5 (3.9)	3 (1.8)	0.298
OCT	16 (5.4)	10 (7.8)	6 (3.6)	0.110

FMD = fibromuscular dysplasia; IVUS = intravascular ultrasound; LAD = left anterior descending (coronary artery); NSTEMI = non-ST-segment elevation myocardial infarction; OCT = optical coherence tomography; PsA = psoriatic arthritis; RA = rheumatoid arthritis; SCAD = spontaneous coronary artery dissection; SLE = systemic lupus erythematosus; STEMI = ST-segment elevation myocardial infarction.

a In women only.

### Clinical characteristics in SCAD patients with and without FMD

Table 1 summarizes the demographic and clinical characteristics stratified by FMD status. SCAD patients with concomitant FMD were significantly older (54.9 ± 9.9 vs 51.7 ± 12.0 years; *P* = 0.015) and more often female (98.4% vs 89.3%; *P* = 0.002) compared with those without FMD. A history of coronary artery disease was less frequent among patients with FMD (18.8% vs 31.0%; *P* = 0.017), whereas a history of migraines was more common (21.1% vs 11.9%; *P* = 0.032). No significant differences were observed between groups in predisposing conditions, precipitating stressors, or clinical presentation. As shown in Supplemental Table 1, initial management strategies did not differ significantly between groups.

### Obstetric and gynecological history in female SCAD patients with and without FMD

Supplemental Table 2 presents obstetric and gynecological characteristics in female patients with SCAD by FMD status. In the overall (unstratified) cohort, 58.6% were postmenopausal, 4.7% had used oral contraception before myocardial infarction, 8.7% had undergone hysterectomy, 3.6% had preeclampsia, 2.9% had endometriosis, and 2.9% had received fertility treatment. These characteristics were largely similar across FMD groups, except for abnormal uterine bleeding, which was more frequently reported in patients with FMD (4.0% vs 0.0%; *P* = 0.019).

### Psychological distress in SCAD patients with and without awareness of FMD diagnosis

**Figure 1:**
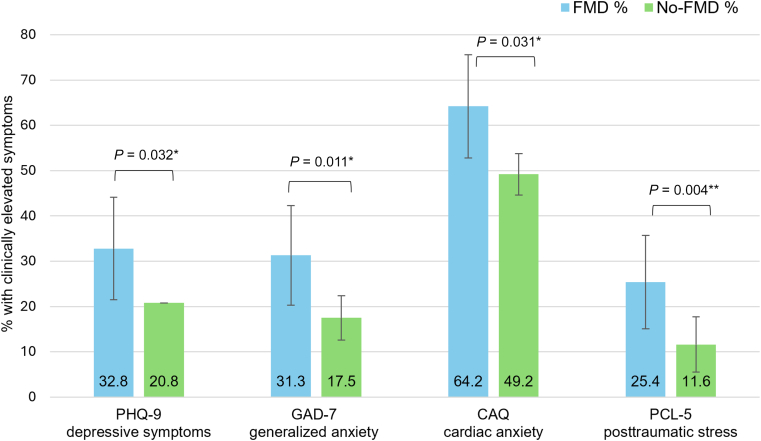
**Psychological Distress in Spontaneous Coronary Artery Dissection With vs Without Awareness of Fibromuscular Dysplasia** Percentages meeting thresholds for clinically significant symptoms were higher among those with a known FMD diagnosis for depression, generalized anxiety, cardiac anxiety, and posttraumatic stress. ∗∗*P* < 0.01, ∗*P* < 0.05. CAQ = Cardiac Anxiety Questionnaire; FMD = fibromuscular dysplasia; GAD-7 = Generalized Anxiety Disorder-7; PCL-5 = Posttraumatic Stress Disorder Checklist for DSM-5; PHQ-9 = Patient Health Questionnaire-9.

At the time of psychological questionnaire completion, 67 SCAD patients (20.6%) reported having been screened for and diagnosed with FMD. Supplemental Table 3 summarizes the mean symptom scores and clinical thresholds for clinically elevated psychological distress. Compared with participants without a known FMD diagnosis, those with awareness of FMD reported significantly higher symptom scores of generalized anxiety (GAD-7: 6.4 ± 5.9 vs 4.7 ± 4.9; *P* = 0.028), cardiac anxiety (CAQ: 29.9 ± 13.1 vs 26.0 ± 12.3; *P* = 0.023), and post-traumatic stress (PCL-5: 17.2 ± 15.4 vs 13.3 ± 14.0; *P* = 0.048). Furthermore, as displayed in Figure 1 and the Central Illustration, patients with SCAD aware of FMD were significantly more likely to have clinically elevated scores across all measures, including depression (32.8% vs 20.8%; *P* = 0.032), generalized anxiety (31.3% vs 17.4%; *P* = 0.011), cardiac anxiety (64.2% vs 49.2%; *P* = 0.031), and post-traumatic stress symptoms (25.4% vs 11.6%; *P* = 0.004).

**Central Illustration:**
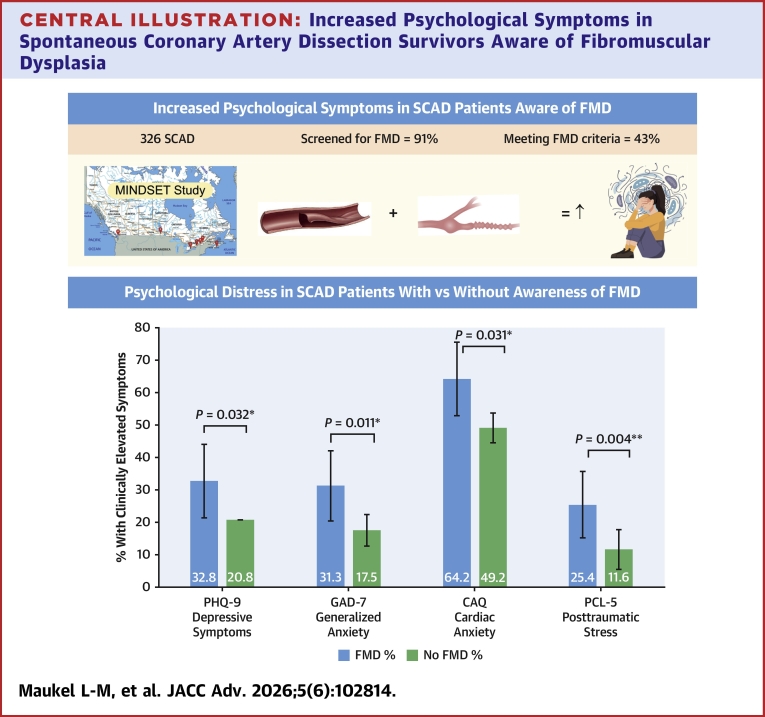
**Increased Psychological Symptoms in Spontaneous Coronary Artery Dissection Survivors Aware of Fibromuscular Dysplasia** Among 326 participants (93% female; mean age 53 years), 91% underwent FMD screening and 43% were diagnosed. Those patients aware of an FMD diagnosis had greater psychological distress and distinct clinical characteristics vs those without known FMD. ∗∗*P* < 0.01, ∗*P* < 0.05. CAQ = Cardiac Anxiety Questionnaire; FMD = fibromuscular dysplasia; GAD-7 = Generalized Anxiety Disorder-7; PCL-5 = Post-traumatic Stress Disorder Checklist; PHQ-9 = Patient Health Questionnaire-9; SCAD = spontaneous coronary artery dissection.

Sensitivity analyses (Supplemental Table 4) in patients with angiographically confirmed FMD (N = 128) confirmed our main findings: patients aware of their diagnosis showed significantly higher generalized and cardiac-specific anxiety, with similar directions observed for depressive and posttraumatic stress symptoms.

Table 2 shows multivariable regression analyses for psychological distress. FMD awareness was significantly associated with higher scores in generalized anxiety (*B* = 1.60, *P* = 0.019) and cardiac anxiety symptoms (*B* = 4.13, *P* = 0.014), after adjusting for sex, age, and pre-SCAD mental health diagnosis.

**Table 2:** Multiple Regression Models of Psychological Distress

	PHQ-9				GAD-7			
	*B* (SE)	95% CI	*t*	*P* Value	*B* (SE)	95% CI	*t*	*P* Value
Depressive Symptoms (PHQ-9)	0.96 (0.73)	−0.47 to 2.39	1.32	0.187	1.60 (0.68)	0.27-2.93	0.68	0.019
Anxiety symptoms (GAD-7)	3.16 (1.14)	0.92-5.40	2.76	0.006	1.63 (1.07)	−0.46 to 3.72	1.07	0.127
Cardiac anxiety (CAQ)	−0.11 (0.03)	−0.17 to −0.06	−4.28	<0.001	−0.11 (0.02)	−0.16 to −0.06	0.02	<0.001
Post-traumatic stress (PCL-5)	2.35 (0.66)	1.05-3.64	3.56	<0.001	1.86 (0.62)	0.65-3.07	0.62	0.003
*R*^*2*^/*R*^*2*^ adjusted		0.12/0.11			0.11/0.10			

	CAQ				PCL-5			
	B (SE)	95% CI	*t*	*P* Value	B (SE)	95% CI	*t*	*P* Value
Depressive Symptoms (PHQ-9)	4.13 (1.68)	0.84-7.41	2.46	0.014	3.44 (1.91)	−0.30 to 7.17	1.80	0.071
Anxiety symptoms (GAD-7)	6.37 (2.64)	1.20-11.55	2.41	0.016	6.47 (2.99)	0.60-12.33	2.16	0.031
Cardiac anxiety (CAQ)	−0.23 (0.06)	−0.35 to −0.11	−3.72	<0.001	−0.32 (0.07)	−0.45 to −0.18	−4.59	<0.001
Post-traumatic stress (PCL-5)	2.31 (1.52)	−0.68 to 5.29	1.51	0.130	5.86 (1.73)	2.46-9.26	3.38	<0.001
R^2^/R^2^ adjusted	0.09/0.07				0.12/0.11			

Results are pooled estimates from multiple imputation. Unstandardized regression coefficients (*B*) are reported instead of standardized betas (*β*), each *B* represents the expected change in the outcome score for a one-unit increase in the predictor. Outcomes score ranges are PHQ-9: 0 to 27, GAD-7: 0 to 21, CAQ: 0 to 72, PCL-5: 0 to 80. FMD awareness: 0 = no, 1 = yes; Sex: 0 = male, 1 = female; Pre-SCAD mental health diagnosis: 0 = no, 1 = yes.

CAQ = Cardiac Anxiety Questionnaire for cardiac anxiety; GAD-7 = Generalized Anxiety Disorder-7 for generalized anxiety; PCL-5 = Post-traumatic Stress Disorder Checklist for DSM-5 for post-traumatic stress symptoms; PHQ-9 = Patient Health Questionnaire-9 for depressive symptoms; other abbreviations as in Table 1.

## Discussion

This multicenter study examined demographic, clinical, and psychological differences between SCAD patients with and without concomitant FMD. Three key findings were identified: 1) among patients with SCAD who underwent FMD screening, 43% were found to have concomitant FMD; 2) patients with both SCAD and FMD differed from those without FMD on several key demographic and clinical characteristics, including being older, more likely to be female, and having a history of migraines; and 3) patients who were aware of their FMD diagnosis reported significantly higher levels of anxiety, cardiac anxiety, and post-traumatic stress symptoms compared with those without a known FMD diagnosis. Multiple variable regression supported these findings for generalized anxiety and cardiac anxiety. Together, these findings provide invaluable insight, particularly regarding the heightened psychological burden associated with a concomitant FMD diagnosis. Future treatment programs and research for patients with SCAD should explicitly incorporate these findings to better address the mental health needs of this population.

Among our patients with SCAD who underwent FMD screening, FMD was detected in nearly half (43%); comparable to studies using similar screening modalities.^27^^,^^28^ Reported prevalence in the literature, however, varies widely (17% to 86%),^11^ likely reflecting differences in screening protocol, numbers of vascular beds assessed, and imaging modalities used.^11^ Currently suggested screening methods include catheter-based angiography or CT/MR angiography.^11^ Catheter-based angiography of bilateral iliofemoral and renal arteries appear more sensitive for detecting FMD,^29^ with studies using this approach often reporting prevalence above 70%.^10^^,^^12^ However, transradial access is now recommended for coronary angiography in ACSs, due to reduced access-site bleeding and vascular complication.^30^ As such, catheter angiography of the iliofemoral and renal arteries is less frequently performed during coronary angiography and may represent a missed diagnostic opportunity. Given the high prevalence of concomitant FMD, implementing routine, timely, and efficient screening pathways is essential for optimal management.

FMD investigation in this study was not mandated but left to the discretion of treating physicians. Notably, almost 10% of patients did not have FMD screening results available at study closure at a mean of 17 months post-SCAD. Experts recommend head-to-pelvis vascular imaging for all SCAD patients to detect extra-coronary abnormalities requiring surveillance or treatment; however, contemporary practice appears to lag behind.^1^ A cohort study from a tertiary cardiac care center, for example, reported that fewer than 20% of SCAD patients underwent comprehensive screening.^31^ Our results far surpass this rate, likely due to our recruitment from large cardiovascular centers with SCAD experts who are keenly aware of the possibly of comorbidity of SCAD and FMD and may be more likely to refer for assessment. These findings underscore the need to optimize FMD screening strategies, as well as provide physician education and improved care coordination. Adoption of imaging protocols that allow evaluation of multiple vascular beds in a single session may further enhance screening rates.^32^

Our analysis of patients with SCAD by FMD status showed significant demographic and clinical differences; these findings add to the limited published literature (n = 3 studies). Consistent with prior reports,^12^, ^13^, ^14^ our study patients with FMD were more often female and generally older compared with those without FMD. Specifically, FMD was more frequently documented among postmenopausal women, a finding, whereas not statistically significant, was also observed in a recent French study.^14^ The higher prevalence of FMD in older and postmenopausal patients may reflect an age-related penetrance of the disease, potentially related to hormonal changes and other age-associated vascular processes. Alternatively, vascular abnormalities may already exist but remain below the detection threshold of angiography.^13^^,^^14^

Our clinical data also showed a higher prevalence of migraines among SCAD patients with FMD. A prior report from the iSCAD Registry showed that SCAD patients with migraines were more likely to have internal carotid artery FMD (33.8% vs 23.8%),^33^ supporting a potential shared vascular or connective tissue mechanism, which warrants further investigation.

Reproductive factors are often under-reported in SCAD populations despite their potential clinical relevance. In the present study, we comprehensively captured a range of reproductive variables; however, the small number of events for individual factors limits interpretability. Although abnormal uterine bleeding appeared more frequent among patients with FMD, this finding should be considered hypothesis-generating. Exploratory correlation analyses also suggested that hysterectomy was associated with higher depressive symptoms and generalized anxiety in this SCAD cohort. Overall, these results highlight the importance of systematically collecting reproductive history in larger, registry-based SCAD cohorts.

Psychological outcomes differed significantly between SCAD patients with or without a known FMD diagnosis. Those with awareness of a FMD diagnosis reported higher levels of distress, including generalized anxiety, cardiac anxiety, and post-traumatic stress, with a greater proportion meeting clinically elevated thresholds indicative of probable mental health diagnoses. For example, a probable PTSD diagnosis was approximately twice as common in the FMD group compared with those without known FMD. Cardiac anxiety, particularly the attention subscale, which reflects heightened symptom monitoring and vigilance, was also significantly elevated in the FMD group.

Thus, simply the awareness of a concomitant FMD diagnosis may itself influence psychological burden in patients with SCAD. This is consistent with previous research showing that patients with multiple chronic conditions are at an increased risk for depression, anxiety, and stress-related symptoms, with the risk rising as the number of comorbidities increases.^34^ Although multimorbidity in general contributes to distress, FMD appears uniquely impactful in patients with SCAD, as it directly targets their most salient concern, the fear of recurrence, and imposes concrete lifestyle restrictions (eg, limitations on exercise).^35^^,^^36^ These factors specifically exacerbate anxiety and distress in ways that general multimorbidity does not. Given the high co-occurrence of SCAD and FMD, this comorbidity is particularly relevant to patients’ lived experience and clinically important, as mental health issues have been linked to higher rates of major adverse cardiac events in cardiac populations.^37^^,^^38^

Despite numerous reports advocating for integrated psychosocial support within cardiac rehabilitation,^11^^,^^39^ no formal guidelines currently exist, and patients with SCAD frequently report feeling unsupported during recovery.^40^^,^^41^ The concomitant diagnosis of FMD in our population showing increased psychological distress further emphasizes the importance of mental health strategies in this high-risk population.

## Clinical implications

Our findings have important clinical implications. 1) All patients with SCAD should be screened for FMD;^1^^,^^31^ however, the optimal timing of this screening remains uncertain. Systematic implementation and evaluation of efficient screening protocols are essential and may also help identify patients at increased risk for psychological distress. 2) Early, routine, and repeated mental health screening should likewise be prioritized for all patients with SCAD. Depression, anxiety, cardiac anxiety, and post-traumatic stress symptoms should be assessed at multiple time points to capture potential symptom fluctuations, particularly after new diagnoses, such as FMD, once screening results become available. 3) Patients with comorbid SCAD and FMD may represent a higher-risk subgroup for psychological distress and could therefore benefit from timely referral to psychological support services. Interventions may include psychoeducation, cognitive-behavioral therapy, and mindfulness-based approaches.^42^, ^43^, ^44^ The nature of distress in this group, particularly fear of recurrence and concerns related to activity restrictions, suggests that some degree of tailoring may be beneficial.

Future research should employ longitudinal designs to clarify whether clinical outcomes and adverse events differ between SCAD patients with and without concomitant FMD diagnoses. Such studies would also help elucidate long-term mental health challenges in this population. In addition, it would be valuable to explore whether patients with FMD report distinct psychological intervention needs, which could ultimately inform the development of tailored psychosocial support strategies.

### Study Limitations

Despite the new insights provided in this study, there are some limitations to consider. First, diagnostic criteria for FMD are prone to interobserver variability on imaging performed, and variability in performing diagnostic testing. For example, intracoronary imaging to examine coronary FMD is rarely conducted.^45^ Thus, FMD is likely underdiagnosed in our cohort of patients, as not all patients underwent screening, and screening modalities such as CT and MR angiography have low sensitivities compared to catheter angiography. Reliability data for the diagnosis of SCAD and FMD, as well as for chart review abstraction, were not collected; however, these processes followed routine clinical and research protocols.

Second, mental health outcomes were assessed at a single time point. Assessing patients both before and after receiving an FMD diagnosis would provide a clearer understanding of the impact of the additional diagnosis on mental health in SCAD.

Third, given the number of statistical tests conducted, there is an increased risk of type I error. Although many of the examined variables are clinically and statistically interrelated, and strict correction methods (eg, Bonferroni) may be overly conservative in this context, the findings should be interpreted with caution. Emphasis was therefore placed on effect sizes and CIs.

Finally, clinical outcomes or major adverse cardiovascular events were not analyzed. Future prospective studies should investigate this relationship, as existing evidence,^4^^,^^46^ albeit inconsistent,^6^^,^^17^ suggests that the co-occurrence of FMD and SCAD may be associated with worse outcomes following SCAD.

## Conclusions

FMD is highly prevalent among patients with SCAD. Certain demographic and clinical characteristics differ between SCAD patients with and without FMD. SCAD patients with awareness of concomitant FMD report substantially higher levels of psychological distress highlighting the importance of early screening for both FMD and mental health concerns in this population. Tailored interventions for this high-risk subgroup are warranted.

Perspectives **COMPETENCY IN PATIENT CARE AND PROCEDURAL SKILLS:** All patients with SCAD should be screened for FMD. Although screening is increasingly implemented, the optimal timing remains uncertain. Implementation of systematic protocols may also help identify patients at higher psychological risk. Clinicians should recognize the disclosure of an FMD diagnosis as a potential psychological stressor and incorporate anticipatory psychological assessment, patient-centered communication, and early mental health support into routine care and follow-up.

**TRANSLATIONAL OUTLOOK:** Future research should use longitudinal designs to determine whether clinical outcomes and adverse events differ between SCAD patients with and without FMD and to clarify long-term mental health trajectories. Studies should also evaluate optimal strategies for communicating FMD diagnoses and whether structured psychoeducation or early psychological interventions at the time of diagnosis can mitigate subsequent distress. These insights could inform the development of tailored, interdisciplinary care pathways for this high-risk subgroup of patients with SCAD.

## Funding support and author disclosures

This work was supported by a 10.13039/501100000024Canadian Institutes of Health Research (CIHR) Project Grant Program (180593, PI: Tulloch). Dr. Madan is supported by the Heart & Stroke Foundation Polo Chair in Cardiology at the University of Toronto. Dr. Saw has salary support from Michael Smith Foundation for Health Research (10.13039/501100000245MSFHR), and funding from 10.13039/501100000024CIHR and Heart & Stroke Foundation. All other authors have reported that they have no relationships relevant to the contents of this paper to disclose.
