# Supplementary material for: Double Trouble: Clinical and Psychological Characteristics in SCAD With and Without Fibromuscular Dysplasia
Source: JACC Adv. 2026 Jun 17;5(6):102814. doi: 10.1016/j.jacadv.2026.102814 (PMC13309305; doi:10.1016/j.jacadv.2026.102814)
Supplement: Supplemental Material [file mmc1.pdf]

Supplemental Appendix

Title: Double Trouble — Clinical and Psychological Characteristics in SCAD With and Without Fibromuscular Dysplasia

Authors: Lisa-Marie Maukel, Laurie-Anne Boivin-Proulx, Jacqueline Saw, Mina Madan, Shuangbo Liu, Thais Coutinho, Sharon Mulvagh, Christine Pacheco, Karen Bouchard, Jennifer Reed, Luise Sun, Amélie Paquin, Nadia Lappa, Derek So & Heather Tulloch

Table of Content

Table S1. Patient management in SCAD with vs. without FMD.

Table S2. Obstetric and gynecological history in female SCAD patients with vs. without FMD.

Table S3. Psychological distress in SCAD with vs. without awareness of FMD diagnosis

Table S4. Sensitivity Analysis. Psychological distress among patients with confirmed FMD diagnosis: Comparison of those with versus without awareness of FMD diagnosis.

Table S1. Patient management in SCAD with vs. without FMD.

	**Overall (*n* = 296)**	**FMD (*n* = 128)**	**No-FMD (*n* = 168)**	***p*-value**
**Initial management**				.627
POBA, *n* (%)	12 (4.1)	4 (3.2)	8 (4.8)	
DES, *n* (%)	23 (7.9)	12 (9.5)	11 (6.6)	
CABG, *n* (%)	3 (1.0)	1 (0.8)	2 (1.2)	
Conservative, *n* (%)	240 (82.2)	105 (83.3)	135 (81.3)	
**Medical therapy received at discharge**				
ASA, *n* (%)	264 (91.0)	117 (93.6)	147 (89.1)	.183
P2Y12 inhibitor, *n* (%)	63 (21.7)	26 (20.8)	37 (22.4)	.740
Oral anticoagulant, *n* (%)	9 (3.1)	4 (3.2)	5 (3.0)	1.000
Beta-blocker, *n* (%)	272 (92.8)	121 (95.3)	151 (91.0)	.156
Calcium channel blockers, *n* (%)	46 (15.9)	23 (18.4)	23 (13.9)	.302
ACEI/ARB, *n* (%)	122 (42.1)	53 (42.4)	69 (41.8)	.921
SGLT2 inhibitor, *n* (%)	8 (2.7)	1 (0.8)	7 (4.2)	.144
GLP1 agonist, *n* (%)	4 (1.4)	1 (0.8)	3 (1.8)	.636
Nitrates, *n* (%)	16 (5.5)	7 (5.6)	9 (5.5)	.967
Diuretic, *n* (%)	16 (5.5)	6 (4.8)	10 (6.1)	.630
Statin, *n* (%)	91 (31.4)	36 (28.8)	55 (33.3)	.410

Note. SCAD, spontaneous coronary artery dissection; FMD, fibromuscular dysplasia; POBA, plain old balloon angioplasty; DES, drug-eluting stent; CABG, coronary artery bypass grafting; ASA, acetylsalicylic acid; ACEI, angiotensin-converting enzyme inhibitor; ARB, angiotensin receptor blocker; SGLT2 inhibitor, sodium-glucose cotransporter 2 inhibitor; GLP-1 agonist, glucagon-like peptide-1 receptor agonist.

Table S2. Obstetric and gynecological history in female SCAD patients with vs. without FMD.

Condition, *n* (%)	Overall (*n* = 276)	FMD (*n* = 125)	No-FMD (*n* = 146)	*p*-value
Hysterectomy	24 (8.7)	13 (10.3)	11 (7.3)	.381
Endometriosis	8 (2.9)	2 (1.6)	6 (4.0)	.297
Gestational hypertension	5 (1.8)	1 (0.8)	4 (2.7)	.380
Gestational diabetes	6 (2.2)	2 (1.6)	4 (2.7)	.691
Preeclampsia	10 (3.6)	6 (4.8)	4 (2.7)	.520
Intrauterine growth restriction	0 (0.0)	0 (0.0)	0 (0.0)	-
Premature birth	3 (1.1)	1 (0.8)	2 (1.3)	1.000
Placenta abruptio	1 (0.4)	1 (0.8)	0 (0.0)	.457
Stillbirth/fetal demise	6 (2.2)	1 (0.8)	5 (3.3)	.224
Fertility treatment	8 (2.9)	5 (4.0)	3 (2.0)	.476
Pregnancy following fertility treatment	3 (1.1)	2 (1.6)	1 (0.7)	.594
Polycystic ovarian syndrome	7 (2.5)	5 (4.0)	2 (1.3)	.252
Abnormal uterine bleeding	5 (1.8)	5 (4.0)	0 (0.0)	**.019**
Catamenial angina	0 (0.0)	0 (0.0)	0 (0.0)	-
Oral contraception pre-myocardial infarction	13 (4.7)	6 (4.8)	7 (4.7)	.970
Post-menopausal status	143 (58.6)	72 (64.9)	71 (53.4)	.070
Menopausal hormonal therapy	5 (1.8)	4 (3.2)	1 (0.7)	.182

Note. All variables are binary (0 = No, 1 = Yes); percentages reflect “Yes” responses. SCAD = spontaneous coronary artery dissection; FMD = fibromuscular dysplasia.

Table S3. Psychological distress in SCAD with vs. without awareness of FMD diagnosis

	**Overall (*n* = 326)**	**FMD (*n* = 67)**	**No-FMD (*n* = 259)**	***p*-value**
PHQ-9, *M(SD)*	6.3 (5.5)	7.2 (6.4)	6.0 (5.3)	.180
PHQ ≥ 10, *n* (%)	76 (23.3)	22 (32.8)	54 (20.8)	**.032**
GAD7, *M(SD)*	5.1 (5.1)	6.4 (5.9)	4.7 (4.9)	**.028**
GAD-7 ≥10, *n* (%)	66 (20.2)	21 (31.3)	45 (17.5)	**.011**
CAQ total, *M(SD)*	26.8 (12.5)	29.9 (13.1)	26.0 (12.3)	**.023**
CAQ ≥ 26, *n* (%)	172 (52.8)	42 (64.2)	128 (49.2)	**.031**
CAQ avoidance, *M(SD)*	1.6 (1.0)	1.8 (1.0)	1.5 (1.0)	.086
CAQ attention, *M(SD)*	1.2 (0.7)	1.4 (0.7)	1.2 (0.7)	**.037**
CAQ fear, *M(SD)*	1.6 (0.8)	1.8 (0.9)	1.6 (0.8)	.063
PCL-5, *M(SD)*	14.1 (14.4)	17.2 (15.5)	13.3 (14.0)	**.048**
PCL-5 ≥ 31, *n* (%)	47 (14.4)	17 (25.4)	30 (11.6)	**.004**

Note. Patients who self-reported a diagnosis of FMD when completing the questionnaires (n=67) were compared to those who did not report such a diagnosis (n=259). Imputed, pooled values; SCAD, spontaneous coronary artery dissection; FMD, fibromuscular dysplasia; PHQ-9, Patient Health Questionnaire-9 for depressive symptoms; GAD-7, Generalized Anxiety Disorder-7 for generalized anxiety; CAQ, Cardiac Anxiety Questionnaire for cardiac anxiety; PCL-5, PTSD Checklist for DSM-5 for posttraumatic stress symptoms.

Table S4. Sensitivity Analysis. Psychological distress among patients with confirmed FMD diagnosis: Comparison of those with versus without awareness of FMD diagnosis.

	**Overall (*n* = 128)**	**FMD (*n* = 63)**	**No-FMD (*n* = 65)**	***p*-value**
PHQ-9, *M(SD)*	6.9 (6.3)	7.4 (6.5)	6.3 (6.0)	.328
PHQ ≥ 10, *n* (%)	39 (30.5)	21 (33.3)	18 (27.7)	.475
GAD7, *M(SD)*	5.5 (5.7)	6.6 (5.8)	4.5 (5.4)	**.047**
GAD-7 ≥10, *n* (%)	32 (25.0)	20 (31.7)	12 (18.5)	**.041**
CAQ total, *M(SD)*	27.5 (13.2)	30.3 (12.9)	24.8 (13.1)	**.019**
CAQ ≥ 26, *n* (%)	72 (56)	42 (66.7)	30 (46.2)	**.014**
CAQ avoidance, *M(SD)*	1.7 (1.0)	1.8 (1.0)	1.5 (0.9)	.153
CAQ attention, *M(SD)*	1.2 (0.8)	1.4 (0.7)	1.1 (0.7)	**.012**
CAQ fear, *M(SD)*	1.6 (0.9)	1.8 (0.9)	1.5 (0.9)	**.037**
PCL-5, *M(SD)*	15.2 (15.2)	17.6 (15.1)	12.9 (15.2)	.084
PCL-5 ≥ 31, *n* (%)	25 (19.5)	16 (25.4)	9 (13.8)	.231

Note. Among patients with angiographically confirmed FMD diagnoses at study completion (N = 128), those who self-reported a diagnosis of FMD when completing the questionnaires (n = 63) were compared to those who did not report a diagnosis (n = 65). Imputed, pooled values; SCAD, spontaneous coronary artery dissection; FMD, fibromuscular dysplasia; PHQ-9, Patient Health Questionnaire-9 for depressive symptoms; GAD-7, Generalized Anxiety Disorder-7 for generalized anxiety; CAQ, Cardiac Anxiety Questionnaire for cardiac anxiety; PCL-5, PTSD Checklist for DSM-5 for posttraumatic stress symptoms.
